# Does Improved Continuity of Primary Care Affect Clinician–Patient Communication in VA?

**DOI:** 10.1007/s11606-013-2633-8

**Published:** 2013-09-26

**Authors:** David A. Katz, Kim McCoy, Mary Vaughan Sarrazin

**Affiliations:** 1 From the VISN 23 Patient Aligned Care Team (PACT) Demonstration Laboratory, Iowa City VA Medical Center, University of Iowa, Iowa City, IA USA; 2 Department of Medicine, University of Iowa, Iowa City, IA USA; 3 Department of Epidemiology, University of Iowa College of Public Health, Iowa City, IA USA; 4 Comprehensive Access & Delivery Research and Evaluation (CADRE) Center, VA Iowa City Health Care System (152), Iowa City, IA USA

**Keywords:** continuity of care, interpersonal communication, shared decision making, primary care

## Abstract

**BACKGROUND:**

Recent changes in health care delivery may reduce continuity with the patient’s primary care provider (PCP). Little is known about the association between continuity and quality of communication during ongoing efforts to redesign primary care in the Veterans Administration (VA).

**OBJECTIVE:**

To evaluate the association between longitudinal continuity of care (COC) with the same PCP and ratings of patient–provider communication during the Patient Aligned Care Team (PACT) initiative.

**DESIGN:**

Cross-sectional survey.

**PARTICIPANTS:**

Four thousand three hundred ninety-three VA outpatients who were assigned to a PCP, had at least three primary care visits to physicians or physician extenders during Fiscal Years 2009 and 2010 (combined), and who completed the Survey of Healthcare Experiences of Patients (SHEP) following a primary care visit in Fiscal Year (FY)2011.

**MAIN MEASURES:**

Usual Provider of Continuity (UPC), Modified Modified Continuity Index (MMCI), and duration of PCP care were calculated for each primary care patient. UPC and MMCI values were categorized as follows: 1.0 (perfect), 0.75–0.99 (high), 0.50–0.74 (intermediate), and < 0.50 (low). Quality of communication was measured using the four-item Consumer Assessment of Healthcare Providers and Systems-Health Plan program (CAHPS-HP) communication subscale and a two-item measure of shared decision-making (SDM). Excellent care was defined using an “all-or-none” scoring strategy (i.e., when all items within a scale were rated “always”).

**KEY RESULTS:**

UPC and MMCI continuity remained high (0.81) during the early phase of PACT implementation. In multivariable models, low MMCI continuity was associated with decreased odds of excellent communication (OR = 0.74, 95 % CI = 0.58–0.95) and SDM (OR = 0.70, 95 % CI = 0.49, 0.99). Abbreviated duration of PCP care (< 1 year) was also associated with decreased odds of excellent communication (OR = 0.35, 95 % CI = 0.18, 0.71).

**CONCLUSIONS:**

Reduced PCP continuity may significantly decrease the quality of patient–provider communication in VA primary care. By improving longitudinal continuity with the assigned PCP, while redesigning team-based roles, the PACT initiative has the potential to improve patient–provider communication.

## BACKGROUND

Continuity is a core attribute of the patient-centered medical home and of high quality primary care.^1^,^2^ The essence of continuity is that one provider (and his/her team of associated individuals) serves as the patient’s regular source of care over a defined period of time. Continuity of primary care is associated with decreased emergency deparntment (ED) use and hospitalization^3^–^5^ and improved patient satisfaction,^6^,^7^ medication adherence,^8^ and delivery of preventive care.^9^,^10^ Duration of primary care provider (PCP) care is also associated with lower costs of inpatient and outpatient care and with a lower risk of hospitalizations.^11^

One mechanism by which continuity may improve quality and reduce unplanned acute care visits is by improving communication. Good communication is a prerequisite for maintaining a long-term, collaborative relationship with patients, and is a key determinant of patient satisfaction.^12^ Patients enjoy being able to communicate their concerns and having a PCP who is willing to talk and to listen.^13^ Patients also value having a PCP who “knows” and respects them,^14^,^15^ which is facilitated by having repeated visits with the same provider. Through a process of shared decision-making, the provider frames and tailors information based on an understanding of the patient’s concerns, beliefs, and expectations.^16^ Patients who feel rushed or ignored, who receive inadequate advice or explanation, and who spend less time with their physicians during routine visits are generally less satisfied and more likely to pursue malpractice litigation.^17^ Yet, there has been relatively little research on the relationship between primary care continuity and the quality of communication.

Within the Veterans Health Administration (VHA), a 3-year plan to transform primary care began in April 2010 with implementation of the patient-centered medical home model, now known as PACT (Patient Aligned Care Team).^18^ Although PCP continuity is a key attribute of the PACT model, implementation of this model requires the transition from traditional primary care (which emphasizes the individual clinician–patient relationship) to multidisciplinary team-delivered care, which may potentially worsen the quality of clinician–patient interactions.^19^ Indeed, recent studies suggest that loss of “patient-connectedness” to the PCP may worsen the quality of care rendered.^10^ The aim of this study is to evaluate the association between longitudinal continuity of primary care and ratings of physician–patient communication within the context of the PACT initiative. In a secondary analysis, we also assess whether low continuity of care is associated with lower ratings of shared decision making.

## METHODS

### Study Patients

We conducted a retrospective cohort study of VA outpatients in a Veterans Integrated Service Network (VISN 23) that serves more than 400,000 enrolled veterans residing in the states of Iowa, Minnesota, Nebraska, North Dakota, and South Dakota. We included patients who satisfied the following criteria: 1) were assigned to a PCP and had at least three primary care visits to physicians or physician extenders during a 2-year follow-back period; and 2) completed the Survey of Healthcare Experiences of Patients (SHEP) following a primary care visit in Fiscal Year (FY) 2011. We excluded patients who had made fewer than three primary care visits to the VA during the 2-year window for two reasons: 1) it is difficult to obtain a meaningful estimate of continuity with such a small number of visits; and 2) we wanted the analysis sample to include regular users of VA primary care. Patients with dementia (based on ICD-9-CM codes 290 and 331 over the prior 24 months, using inpatient and outpatient files) were also excluded.

### Sampling Strategy and Data Collection

**Figure 1:**
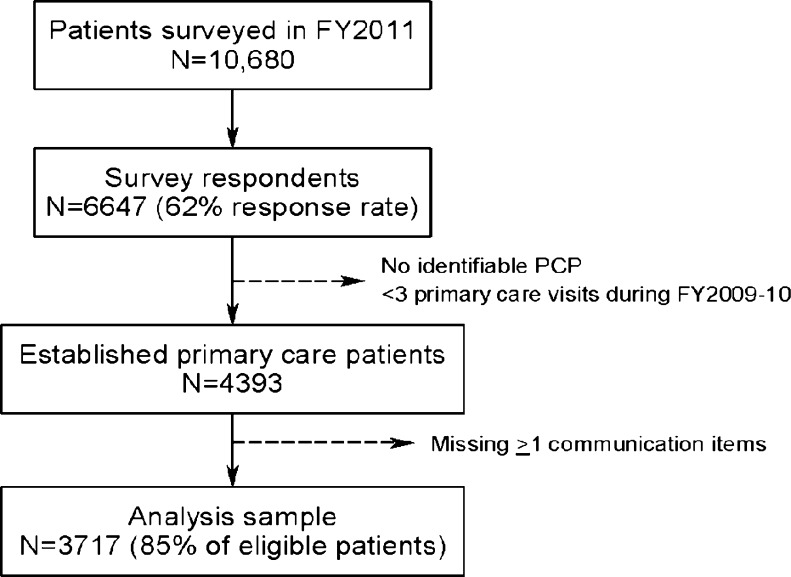
Derivation of analysis sample.

We randomly sampled patients from all primary care clinics in VISN 23 (including four hospital-based clinics that train residents) within two strata: those whose provider participated in a PACT Learning Collaborative and those whose provider did not participate. Based on the Institute for Healthcare Improvement Collaborative model, the VISN 23 Learning Collaborative was intended to equip representatives from PACT teams with the knowledge, skills, and experience to implement medical home principles at their sites and to establish a framework for system-wide learning.^20^ In collaboration with the VA Office of Quality and Performance, a total of 10,680 primary care patients were invited to complete the SHEP survey within a 3-week time period (8/15/11–9/6/11), during the early phase of PACT implementation. Those selected for the survey were sent a presurvey notification letter explaining the goals of the upcoming survey and encouraging the veteran to participate. One week later, the questionnaire was mailed to everyone in the sample; reminder postcards were sent 1 week later.^21^ Sixty-two percent of those invited completed the survey, and 4,393 patients were determined to be eligible for this analysis (Fig. 1).

### Outcome Measures

The SHEP is a 70-item survey that is designed to evaluate veterans’ satisfaction with VHA ambulatory care and services. The survey includes a four-item communication subscale from the Consumer Assessment of Healthcare Providers and Systems-Health Plan program (CAHPS-HP) and two items that assess shared decision making; the SHEP survey items were not modified or adapted for this study. The communication subscale asks the patient to rate (on a four-point Likert type scale) how often over the prior 12 months his personal VA doctor or nurse: 1) explained things in a way that was easy to understand, 2) listened carefully, 3) showed respect for what he or she had to say, and 4) spent enough time with him or her. The same items have been used to assess interpersonal communication in the Medical Expenditure Panel Survey (MEPS).^22^ Cronbach’s alpha for the CAHPS-HP communication subscale is 0.86.^23^ To assess shared decision making (SDM), respondents who faced a treatment choice were also asked to rate (on a four-point Likert type scale) whether or not a VA doctor or other provider: 1) asked about his or her preferences for treatment (when more than one treatment choice was available), and 2) talked to him or her about the pros and cons of each treatment choice.

### Independent Variables

In this analysis, we focus on the effects of longitudinal continuity with the patient’s assigned PCP, which was calculated by linking data from the 2009 Patient Care Management Module with VA outpatient data sets. Clinic stop codes were used to identify primary care visits during FY 2009 and 2010. Longitudinal continuity was determined using three measures: 1) Usual Provider of Continuity (UPC), which was calculated based on the proportion of primary care visits with the patient’s assigned PCP (visit concentration); 2) Modified Modified Continuity Index (MMCI), which accounts for the number of different primary care providers consulted (visit dispersion); and 3) duration of care with the assigned PCP (longitudinality).^24^ MMCI was calculated using the following formula:

$$ \mathrm{MMCI}=\frac{1-\left(\mathrm{No}.\mathrm{of}\ \mathrm{primary}\ \mathrm{care}\ \mathrm{providers}/\left[\mathrm{No}.\mathrm{of}\ \mathrm{primary}\ \mathrm{care}\ \mathrm{visits}+0.1\right]\right)}{1-\left(1/\left[\mathrm{No}.\mathrm{of}\ \mathrm{primary}\ \mathrm{care}\ \mathrm{visits}+0.1\right]\right)} $$

The MMCI score ranges from 0 (if there is maximum dispersion, and if each visit is to a different provider) to 1 (if every visit is to the same provider). UPC and MMCI were selected because these measures are commonly used by the VHA to monitor continuity nationally. Duration of care relates to the length of the patient–provider relationship and complements visit-based measures of continuity.

We calculated UPC and MMCI values for each eligible VISN 23 primary care patient (on a scale of 0–1, where 1 is perfect continuity), and grouped these values into four categories (similar to the categories used by Rodriguez et al.)^19^: 1.0 (perfect), 0.75–0.99 (high), 0.50–0.74 (medium), and < 0.50 (poor). Duration of care was grouped into the following groups: less than 1 year, 1 year to less than 3 years, 3 years to less than 5 years, 5 years to less than 10 years, and 10 years or more.^11^,^25^ Telephone contacts, home-based contacts, or contacts with a non-PCP were excluded in calculating continuity.

### Statistical Analysis

We compared patient characteristics across all categories of longitudinal continuity using the chi-squared and analysis of variance tests for dichotomous and continuous variables (Kruskal-Wallis test if not normally distributed).

To identify excellent care based on the interpersonal communication and shared decision making subscales, we used an “all-or-none” scoring strategy: when all items within a subscale were rated “always,” the subscale score was assigned a value of 1 (otherwise 0). We dichotomized both outcomes because we were primarily interested in modeling superior care and because both measures of communication showed large ceiling effects (with distributions that were skewed to the right); a similar approach has been used to model patient satisfaction data.^26^ Multivariable random effects logistic regression models were used to predict excellent care during FY 2011, after controlling for sociodemographics (age, gender, race, marital status, VA income category), disability status, chronic medical and psychiatric comorbidities, number of primary care clinic visits during FY 2009 and 2010, PCP participation in a PACT Learning Collaborative (yes or no), and usual site of care (modeled as a random effect).

To adjust for comorbidity, we used ICD-9-CM codes in outpatient and inpatient administrative data (during FY 2009 and 2010) to capture 17 medical comorbidities^27^ and five psychiatric comorbidities (depressive disorders, anxiety disorders, post-traumatic stress disorder, bipolar disorders, and psychotic disorders);^28^ each comorbidity was dichotomized as present or absent. We used both outpatient and inpatient VA data for comorbidity adjustment, based on empiric data that show improved prediction of 1-year mortality when both data sources are included.^29^,^30^ Comorbid conditions that affected less that 1 % of the analysis sample were excluded from multivariable models.

All analyses were performed using SAS for Windows, version 9.3 (SAS Institute, Cary, NC). All tests were two-sided and a *p* value of ≤ 0.05 was defined as statistically significant. We did not impute missing values.

## RESULTS

Of the 6,647 patients in VISN 23 who completed the SHEP survey (62 % response rate), 4,393 were eligible for this analysis (Fig. 1). Of these patients, 3,717 had completed all of the CAHPS-HP items (85 % of those eligible) and 1,948 patients completed the SDM survey items (96 % of these patients reported having been faced with a treatment choice over the prior 12 months). The mean UPC and MMCI scores for patients in the analysis sample were nearly identical: 0.81 (sd = 0.25) and 0.81, sd = 0.24), respectively; thus, we focus on UPC results below. The median duration of care with the assigned PCP was 3.1 years (IQR = 2.1, 5.0). Patients in the four UPC categories had similar demographic and clinical characteristics, except for the following: 1) only 39 % of patients with perfect UPC had any service connected disability, compared with 49 % with low UPC (defined as < 0.50), and 2) those with perfect UPC were also less likely to have been diagnosed with depression (12 vs. 17 %, respectively) (Table 1).

**Table 1:** Patient Characteristics Across Categories of Usual Provider Continuity

	Perfect (*N* = 1900)	High (*N* = 718)	Intermediate (*N* = 678)	Low (*N* = 421)
Age, mean (sd)	71 (10)	69 (10)	68 (10)	67 (11)
Gender, % male	98	97	96	98
Married, %	71	69	70	68
Indigent (low income status), %	22	20	20	22
Disability status, 50 % or greater	14	26	25	25
Comorbid medical conditions, %*				
Myocardial infarction	4	5	5	4
Congestive heart failure	9	12	11	13
Peripheral vascular disease	10	11	13	13
Cerebrovascular disease	8	11	9	8
Chronic obstructive pulmonary disease	23	28	25	29
Liver disease (any severity level)	1	1	1	< 1
Diabetes mellitus	39	46	38	40
Moderate or severe renal disease	8	11	10	14
Diabetes with end organ damage	12	18	13	17
Any tumor (including leukemia/lymphoma)	14	15	18	15
Rheumatologic disease	2	2	2	1
Peptic ulcer	1	3	2	1
Comorbid psychiatric conditions, %				
Depressive disorders	12	21	17	17
Anxiety disorders	5	11	10	9
Post-traumatic stress disorder	3	10	10	9
Bipolar disorders	1	2	3	2

*The following comorbid conditions were present in less than 1 % in the analysis sample and are not shown: hemiplegia, metastatic solid tumor, and psychotic disorders

**Figure 2:**
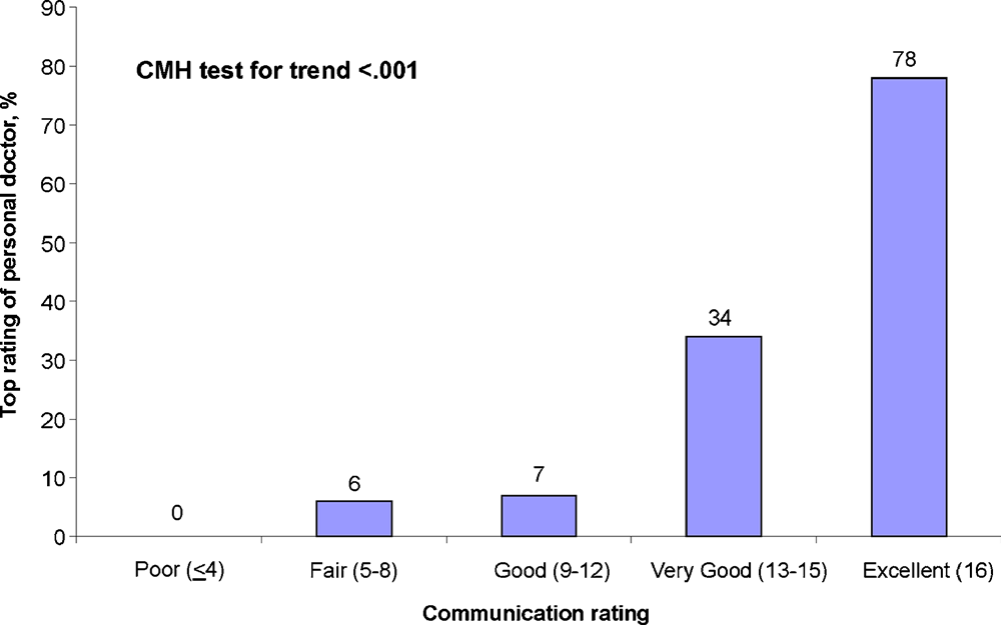
Relationship between CAHPS-HP communication scores and PCP ratings.

The majority of survey respondents (58 %) rated the communication with their PCP as excellent (i.e., all items received a top score). For example, 72 and 74 % of patients indicated that their PCP explained things clearly and “listened carefully” to them, respectively. Internal consistency reliability for the CAHPS-HP communication subscale was high (Cronbach’s alpha = 0.89). Validity is supported by the finding that communication scores were strongly associated with overall PCP ratings (Fig. 2). In addition, we found that only 33 of 157 (21 %) patients who had a complaint about their visit reported excellent interpersonal communication with their PCP.

Fifty-one percent of respondents rated SDM as excellent; 57 % of patients reported that their VA provider had asked about their treatment preferences (when more than one treatment choice was available), and 68 % reported that their VA provider talked with them about the pros and cons of each treatment choice. Cronbach’s alpha of the two-item SDM subscale was 0.74; convergent validity is supported by the finding that the SDM subscale values are significantly correlated with those of the communication subscale (Spearman correlation = 0.47, *p* = 0.01).

In bivariate analysis, there was a positive association between UPC and quality of patient–provider communication: 61 % of patients with perfect continuity of care had excellent ratings of communication (i.e., “always” on all four items), compared to 53 % of those with low continuity (CMH test for trend, *p* = 0.0001); similar results were observed for MMCI continuity (*p* = 0.003) (Table 2). In multivariable random effects models, patients with low UPC continuity were less likely to give excellent ratings of interpersonal communication (OR = 0.80, 95 % CI = 0.64, 1.0). Patients with low MMCI scores were also significantly less lively to give excellent ratings of interpersonal communication (OR = 0.74, 95 % CI = 0.58–0.95) and SDM (OR = 0.70, 95 % CI = 0.49, 0.99). Compared to patients with 10+ years of PCP care, those with PCP relationships less than 1 year were significantly less likely to report superior interpersonal communication (OR = 0.35, 95 % CI = 0.18–0.71); however, there was no significant association between duration of care and SDM (Table 3). Provider participation in a PACT Learning Collaborative was not significantly associated with excellent ratings of interpersonal communication (OR = 1.07, 95 % CI = 0.93, 1.22) or SDM (OR = 0.88, 95 % CI = 0.73–1.06) in models of UPC continuity.

**Table 2:** Quality of Communication as a Function of Longitudinal Continuity (Bivariate Analysis)*

A) UPC and MMCI continuity			
UPC Continuity	Percentage (N)	Communication (% excellent)	SDM (% excellent)
Perfect (1.0)	51 (1,900)	61	51
High (0.75–0.99)	19 (718)	59	53
Intermediate (0.50–0.74)	18 (678)	55	50
Low (< 0.50)	11 (421)	53	50
Test for trend (*p* value)^†^		0.0002	0.72
MMCI Continuity	Percentage (N)	Communication (% excellent)	SDM (% excellent)
Perfect (1.0)	51 (1898)	61	51
High (0.75–0.99)	17 (642)	56	54
Intermediate (0.50–0.74)	23 (873)	58	51
Low (< 0.50)	8 (304)	52	44
Test for trend (*p* value)^†^		0.003	0.42
B) Duration of care with the assigned PCP			
Quartile (years)	Percentage (N)	Communication (% excellent)	SDM (% excellent)
10 or more years	4 (151)	80	60
5–9.9 years	19 (725)	63	54
3–4.9 years	35 (1299)	63	50
1–2.9 years	40 (1497)	57	51
Less than 1 year	1 (45)	43	46
Test for trend (*p* value)^†^		0.20	0.35

*Based on the number of patients with complete data: 3,717 for communication, 1,948 for shared decision making (SDM)

†Cochran-Mantel-Haenszel test for trend

**Table 3:** The Association Between PCP Continuity and Quality of Communication (Multivariable Analysis)

Continuity measure	Adjusted OR (95 % CI)	
	Interpersonal Communication	Shared decision making
UPC		
Perfect (1.0, reference)	1.00	1.00
High (0.75–0.99)	1.02 (0.85, 1.23)	1.08 (0.84, 1.39)
Medium (0.50–0.74)	0.86 (0.71, 1.03)	0.86 (0.67, 1.12)
Low (< 0.50)	0.80 (0.64, 1.00)*	0.86 (0.64, 1.18)
MMCI		
Perfect (1.0, reference)	1.00	1.00
High (0.75–0.99)	0.94 (0.76, 1.16)	1.06 (0.79, 1.40)
Medium (0.50–0.74)	0.96 (0.81, 1.13)	0.96 (0.76, 1.21)
Low (< 0.50)	0.74 (0.58, 0.95)^*^	0.70 (0.49, 0.99)*
Duration of PCP care		
10 or more years (reference)	1.00	1.00
5.0–9.9 years	0.82 (0.56, 1.19)	0.78 (0.45, 1.34)
3.0–4.9 years	0.79 (0.55, 1.14)	0.67 (0.39, 1.14)
1.0–2.9 years	0.72 (0.50, 1.03)	0.68 (0.40, 1.14)
Less than 1 year	0.35 (0.18, 0.71)^*^	0.57 (0.23, 1.43)

*Statistically significant at *p* < 0.05. All models were adjusted for age, gender, race, marital status, VA disability status, disability status, chronic medical comorbidities (Romano), psychiatric comorbidities, number of primary care clinic visits, PCP participation in a PACT Learning Collaborative, and usual site of care (random effect)

## DISCUSSION

Continuity of care is a core metric used by the Veterans Health Administration to monitor the progress of primary care sites as they migrate to the PACT model.^18^ Good patient–provider communication is the cornerstone of relational continuity,^31^ and serves at least six fundamental functions: fostering healing relationships, exchanging information, responding to patients’ emotions, managing uncertainty, making informed decisions, and enabling patient self-management.^32^ Having a provider who is understanding and easy to talk with is highly valued by primary care patients.^33^ In this prospective analysis, we found a dose–response relationship between longitudinal PCP continuity and patient ratings of interpersonal communication. This association was consistent across both measures of visit concentration and dispersion. In addition, we found that patients with increased dispersion of PCP care were less likely to give excellent ratings of shared decision making and that patients with shorter duration of PCP care (less than 1 year) were less likely to report superior interpersonal communication.

This suggests that longitudinal continuity functions as a critical gateway for the delivery of superior interpersonal care.^34^ Indeed, the protective effects of longitudinal continuity on unplanned ED visits and hospitalizations may be mediated in part by improved PCP communication (and its downstream positive effects on adherence to treatment and patient satisfaction). Decreased continuity of care may impact quality of communication through several potential mechanisms: 1) less provider knowledge of the patient’s medical history, 2) less familiarity with the patient’s psychosocial factors, and 3) less trust in the provider. We note, however, that the relationship between continuity of care and quality of communication is likely bidirectional. Specifically, patients who give suboptimal ratings to their PCP on measures of communication may be more likely to switch providers^35^ and have short patient–provider relationships and reduced continuity.^36^

In multivariable analysis, we did not find any association between PCP participation in a PACT Learning Collaborative and quality of communication. One possible explanation of this finding is that the learning sessions focused on team development, care management strategies, and other “back office” functions that may not necessarily lead to perceptible improvements in patients’ ratings of quality. Greater attention to “high touch” dimensions of PACT during training (in contrast to the emphasis placed on “high tech” dimensions)^37^ may have led to a stronger association between PCP participation in the Learning Collaborative and quality of communication.

This study builds on the results of prior studies in primary care practice, most of which used a cross-sectional study design. In one survey of Medicaid patients, increased self-reported continuity of care improved ratings of provider communication and patients’ perception of their ability to influence treatment.^38^ In a study of VA primary care patients, those with high continuity of care reported substantially higher satisfaction with their providers’ communication skills and humanistic qualities, compared to those with low continuity of care.^7^ In a study of patients in a large multi-specialty practice in Massachusetts, visit continuity (measured during the 6 months prior to administration of the Ambulatory Care Experiences Survey) was shown to be positively associated with the quality of clinician–patient communication, especially for those respondents who were in the early stages of the patient–provider relationship and those with worse self-rated health.^19^

Limitations of this analysis warrant further discussion. First, we did not collect SHEP survey data from a comparable sample of primary care patients prior to April, 2010 (before PACT implementation). Second, we lacked data on use of non-VA primary care at the time of this analysis. Of note, this analysis was restricted to regular users of VA primary care; dual users of VA and non-VA care who presented to the VA solely for annual medication refills were excluded. Third, continuity with the assigned PCP did not account for non-face-to-face encounters (e.g., telephone, email); these encounters are not consistently documented in VA records. Fourth, the SHEP survey included only two items on SDM and these items were not specific to the primary care provider. Fifth, global satisfaction with the PCP (used to validate the communication subscale) was based on a single item. Multi-item scales have better measurement characteristics (e.g., content validity, reliability) than single item measures.^39^ Sixth, we used VA administrative data to adjust for comorbid conditions; residual confounding on account of unmeasured comorbidities may still be present. Finally, patients were restricted to a single region of the Department of Veterans Affairs health care system in the upper Midwest. It is unknown whether our findings can be extrapolated to regions with a larger urban and racially diverse patient population.

Several recent changes in health care delivery nationwide, such as the advent of open access scheduling, increased availability of urgent care clinics, growing panel sizes, more providers working part-time, and frequent changes in insurance coverage, have reduced continuity in primary care.^40^–^43^ Efforts to implement medical home models of care may help reverse this trend by improving longitudinal continuity with primary care providers, leading to improved patient–provider communication. Indeed, between FY 2009 and 2012 (approximately 2 years after PACT implementation in VHA), UPC continuity in VISN 23 increased modestly from 0.73 to 0.75 (with a similar improvement in MMCI continuity). Further improvements in care can be realized by delivering instruction on communication skills, including peer evaluation and feedback of patient survey data, to practicing physicians and residents.^44^ Health care managers and clinical leaders who are charged with implementing medical homes must not lose sight of the central importance of longitudinal continuity in creating the conditions for excellent communication, upon which other key goals of the PACT model depend (i.e., promoting goal setting, self management, and respect for patient preferences). Similarly, efforts to improve access to first-contact care and to increase clinic throughput must be closely monitored for their effects on PCP continuity and patient–provider communication. As the medical home model is fully implemented in VA primary care, future research should determine the relationship between emerging measures of team continuity^45^ and the multiple facets of interpersonal care.

## References

[1] Stange KC, Nutting PA, Miller WL (2010). Defining and measuring the patient-centered medical home. J Gen Intern Med.

[2] Institute of Medicine (1996). Primary Care: America’s Health in a New Era.

[3] Wasson JH, Sauvigne AE, Mogielnicki P (1984). Continuity of outpatient medical care in elderly men: a randomized trial. JAMA.

[4] Mainous AG, Gill JM (1998). The importance of continuity of care in the likelihood of future hospitalization: is site of care equivalent to a primary clinician?. Am J Public Health.

[5] Gill JM, Mainous AG, Nsereko M (2000). The effect of continuity of care on emergency department use. Arch Fam Med.

[6] Weyrauch KF (1996). Does continuity of care increase HMO patients’ satisfaction with physician performance?. J Am Board Fam Pract.

[7] Fan VS, Burman M, McDonell MB, Fihn SD (2005). Continuity of care and other determinants of patient satisfaction with primary care. J Gen Intern Med.

[8] Parchman ML, Pugh JA, Noël PH, Larme AC (2002). Continuity of care, self-management behaviors, and glucose control in patients with type 2 diabetes. Med Care.

[9] Cabana MD, Jee SH (2004). Does continuity of care improve patient outcomes?. J Fam Pract.

[10] Atlas SJ, Grant RW, Ferris TG, Chang Y, Barry MJ (2009). Patient–physician connectedness and quality of primary care. Ann Intern Med.

[11] Weiss LJ, Blustein J (1996). Faithful patients: the effect of long-term physician-patient relationships on the costs and use of health care by older Americans. Am J Public Health.

[12] Roberge D, Beaulieu MD, Haddad S, Lebeau R, Pineault R (2001). Loyalty to the regular care provider: patients’ and physicians’ views. Fam Pract.

[13] Pandhi N, Saultz JW (2006). Patients’ perceptions of interpersonal continuity of care. J Am Board Fam Pract.

[14] Gabel LL, Lucas JB, Westbury RC (1993). Why do patients continue to see the same physician?. Fam Pract Res J.

[15] Cheraghi-Sohi S, Hole AR, Mead N (2008). What patients want from primary care consultations: a discrete choice experiment to identify patients’ priorities. Ann Fam Med.

[16] Epstein RM, Fiscella K, Lesser CS, Stange KC (2010). Why the nation needs a policy push on patient-centered health care. Health Aff.

[17] Levinson W, Roter D, Mullooly J, Dull V, Frankel R (1997). Physician–patient communication: the relationship with malpractice claims among primary care physicians and surgeons. JAMA.

[18] Klein S (2011). The Veterans Health Administration: Implementing Patient-Centered Medical Homes in the Nation’s Largest Integrated Delivery System.

[19] Rodriguez HP, Rogers WH, Marshall RE, Safran DG (2007). The effects of primary care physician visit continuity on patients’ experiences with care. J Gen Intern Med.

[20] Solimeo S, Hein M, Paez M, Ono S, Lampman M, Stewart G (2013). Medical homes require more than an EMR and aligned incentives. Am J Manag Care.

[21] Dillman DA (2009). Internet, Mail, and Mixed-Mode Surveys: The Tailored Design Method.

[22] DeVoe JE, Wallace LS, Pandhi N, Solotaroff R, Fryer GE (2008). Comprehending care in a medical home: a usual source of care and patient perceptions about healthcare communication. J Am Board Fam Med.

[23] Hargraves JL, Hays RD, Cleary PD (2003). Psychometric properties of the Consumer Assessment of Health Plans Study (CAHPS) 2.0 Adult Core Survey. Health Serv Res.

[24] Jee SH, Cabana MD (2006). Indices for continuity of care: a systematic review of the literature. Med Care Res Rev.

[25] Love MM, Mainous AG (1999). Commitment to a regular physician: how long will patients wait to see their own physician for acute illness?. J Fam Pract.

[26] Holmboe ES, Arnold GK, Weng W, Lipner R (2010). Current yardsticks may be inadequate for measuring quality improvements from the medical home. Health Aff.

[27] Romano PS, Roos LL, Jollis JG (1993). Adapting a clinical comorbidity index for use with ICD-9-CM administrative data: differing perspectives. J Clin Epidemiol.

[28] Abrams TE, Vaughan-Sarrazin M, Rosenthal GE (2009). Psychiatric comorbidity and mortality after acute myocardial infarction. Circ Cardiovasc Qual Outcomes.

[29] Schneeweiss S, Seeger JD, Maclure M, Wang PS, Avorn J, Glynn RJ (2001). Performance of comorbidity scores to control for confounding in epidemiologic studies using claims data. Am J Epidemiol.

[30] Schneeweiss S, Wang PS, Avorn J, Glynn RJ (2003). Improved comorbidity adjustment for predicting mortality in Medicare populations. Health Serv Res.

[31] Saultz JW (2003). Defining and measuring interpersonal continuity of care. Ann Fam Med.

[32] Epstein RM, Street RL (2007). Patient-Centered Communication in Cancer Care: Promoting Healing and Reducing Suffering.

[33] Fletcher RH, O’Malley MS, Earp JA (1983). Patients’ priorities for medical care. Med Care.

[34] Cheraghi-Sohi S, Bower P, Mead N, McDonald R, Whalley D, Roland M (2006). What the are key attributes of primary care for patients? Building a conceptual ‘map’ of patient preferences. Health Expect.

[35] Safran DG, Montgomery JE, Chang H, Murphy J, Rogers WH (2001). Switching doctors: predictors of voluntary disenrollment from a primary physician’s practice. J Fam Pract.

[36] Hjortdahl P, Laerum E (1992). Continuity of care in general practice: effect on patient satisfaction. BMJ.

[37] Ferrante JM, Balasubramanian BA, Hudson SV, Crabtree BF (2010). Principles of the patient-centered medical home and preventive services delivery. Ann Fam Med.

[38] Love MM, Mainous AG, Talbert JC, Hager GL (2000). Continuity of care and the physician-patient relationship: the importance of continuity for adult patients with asthma. J Fam Pract.

[39] Nunnally J (1978). Psychometric Theory.

[40] Flocke SA (1997). Measuring attributes of primary care: development of a new instrument. J Fam Pract.

[41] Moore G, Showstack J (2003). Primary care medicine in crisis: toward reconstruction and renewal. Ann Intern Med.

[42] Salisbury C, Sampson F, Ridd M, Montgomery AA (2009). How should continuity of care in primary health care be assessed?. Br J Gen Pract.

[43] Phan K, Brown S (2009). Decreased continuity in a residency clinic: a consequence of open access scheduling. Fam Med.

[44] Levinson W, Lesser CS, Epstein AM (2010). Developing physician communications skills for patient-centered care. Health Aff.

[45] Uijen AA, Heinst CW, Schellevis FG (2012). Measurement properties of questionnaires measuring continuity of care: a systematic review. PLoS One.

